# Supplementary material for: Diagnostically competitive performance of a physiology-informed generative multi-task network for contrast-free CT perfusion
Source: Front Hum Neurosci. 2026 Jan 26;20:1703004. doi: 10.3389/fnhum.2026.1703004 (PMC12883745; doi:10.3389/fnhum.2026.1703004)
Supplement: Supplementary file 1 [file Data_Sheet_1.pdf]

Supplementary Materials

Diagnostically Competitive Performance of a Physiology-Informed Generative Multi-Task Network for Contrast-Free CT Perfusion

Wasif Khan[a], John Rees[b], Kyle B. See[a], Simon Kato[c], Ziqian Huang[a], Amy Lazarte[a], Kyle Douglas[a], Xiangyang Lou[d], Teng J. Peng[e], Dhanashree Rajderkar[b], Pina Sanelli[f,g,h], Amita Singh[e], Ibrahim Tuna[b], Christina A. Wilson[e], Ruogu Fang[a,i,j,*]

[a] *J. Crayton Pruitt Family Department of Biomedical Engineering, University of Florida, Gainesville, FL, USA*
[b] *Department of Radiology, University of Florida, Gainesville, FL, USA*
[c] *Department of Mathematics and Statistics, University of Florida, Gainesville, FL, USA*
[d] *Department of Biostatistics, University of Florida, Gainesville, FL, USA*
[e] *Department of Neurology, University of Florida, Gainesville, FL, USA*
[f] *Feinstein Institutes for Medical Research, Manhasset, NY, USA*
[g] *Department of Radiology, Donald and Barbara Zucker School of Medicine at Hofstra Northwell, Hempstead, NY, USA*
[h] *Northwell Health, Manhasset, NY, USA*
[i] *Center for Cognitive Aging and Memory, McKnight Brain Institute, University of Florida, Gainesville, FL, USA*
[j] *Department of Electrical and Computer Engineering, University of Florida, Gainesville, FL, USA*

*Corresponding author
Email address: ruogu.fang@bme.ufl.edu (Ruogu Fang)

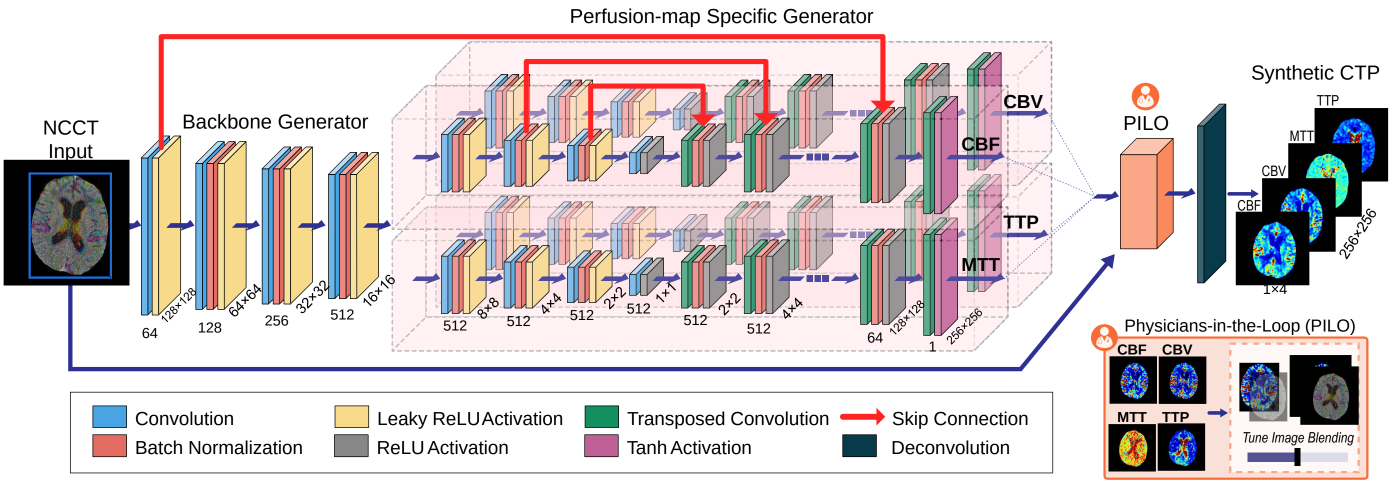

Figure S.1: A diagram of the generator network architecture. The generator follows a modified U-Net architecture, with various network layers connected by skip connections. The first encoding layers share the encoded weights of each map before diverging into multi-task perfusion map generation. The physicians-in-the-loop module comprises the final layers of the architecture in which the NCCT input is concatenated with the initial perfusion output. A final deconvolutional layer is applied to generate the final perfusion map outputs.

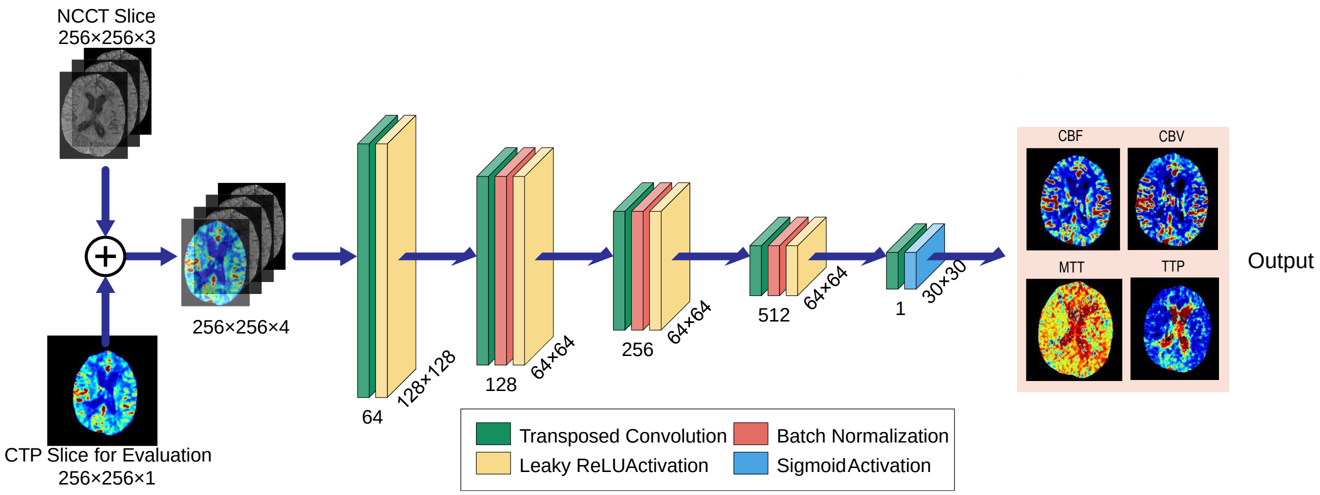

Figure S.2: Discriminator network architecture which is based on the PatchGAN framework, which evaluates the realism of local image patches rather than entire images. The discriminator applies a series of transposed convolutional layers to the concatenated input of the NCCT slice and the CTP slice for evaluation.

Q1. Do you think this is a real CT perfusion map?

1. **Yes**
0. **No**

Q2. For the following questions A-D, make your best efforts to choose "Acceptable" or "Unacceptable." If you choose "Indeterminate," please provide a short explanation of your answer.

a. **What is the diagnostic quality of the CBV maps?**

   1. Acceptable
   0. Indeterminate
   -1. Unacceptable

b. **What is the diagnostic quality of the CBF maps?**

   1. Acceptable
   0. Indeterminate
   -1. Unacceptable

c. **What is the diagnostic quality of the MTT maps?**

   1. Acceptable
   0. Indeterminate
   -1. Unacceptable

d. **What is the diagnostic quality of the TTP maps?**

   1. Acceptable
   0. Indeterminate
   -1. Unacceptable

Q3. What diagnosis would be given to this patient based on their NCCT and CTP imaging?

1. **Normal exam with no perfusion deficit**
2. **Core infarct is less than or equal to 20% of the tissue at risk**
3. **Core infarct is strictly more than 20% of the tissue at risk**
4. **Core infarct is approximately equal to tissue at risk**

Q4. Rate your confidence in the above diagnosis.

1. **Not confident**
2. **Slightly confident**
3. **Somewhat confident**
4. **Fairly confident**
5. **Completely confident**

Figure S.3: The questionnaire provided to evaluators for double-blinded evaluation.

Table S.1: The results of Q1 from the double-blinded questionnaire: “Do you think this is a real CT perfusion map?”. All raters were unable to differentiate between real and synthetic CTP imaging. Overall, both real (RAPID) and synthetic (MAGIC) CTP images were predominantly classified as ”RAPID”— 117 and 118 responses, respectively. This trend held across individual doctors, with the exception of Doctor 7, who more frequently identified images as MAGIC. These findings suggest that raters predominantly treated both real and synthetic CTP maps as real, indicating that MAGIC is capable of generating synthetic CTP maps that closely resemble real ones.

**Doctor**	**RAPID**		**MAGIC**	
	**Is RAPID**	**Is MAGIC**	**Is RAPID**	**Is MAGIC**
1	12	8	15	5
2	19	1	19	1
3	19	1	16	4
4	20	0	18	2
5	20	0	18	2
6	19	1	19	1
7	8	12	13	7
**Sum**	**117**	**23**	**118**	**22**

Table S.2: Response comparison for RAPID and MAGIC CTP on Question 2: "What is the diagnostic quality of this perfusion map?" Counts of Acceptable (Acc), Indeterminate (Indet), and Unacceptable (Unacc) ratings for CBV maps are shown. Most images were rated "Acceptable" for both methods.

**Doctor**	**RAPID CBV**			**MAGIC CBV**		
	Acc	Indet	Unacc	Acc	Indet	Unacc
1	20	0	0	19	1	0
2	20	0	0	18	1	1
3	18	2	0	18	1	1
4	20	0	0	18	2	0
5	20	0	0	17	1	2
6	20	0	0	20	0	0
7	20	0	0	19	0	1
**Sum**	**138**	**2**	**0**	**129**	**6**	**5**

Table S.3: Comparison of individual responses to RAPID and MAGIC CTP for Questions 2: "What is the diagnostic quality of this perfusion map?" The table shows the response counts (Acceptable, Indeterminate, Unacceptable) for CBF maps processed by RAPID and MAGIC. Most images were rated "Acceptable" across both methods, with very few "Indeterminate" or "Unacceptable" responses. Acc = Acceptable, Indet = Indeterminate, Unacc = Unacceptable.

**Doctor**	**RAPID CBF**			**MAGIC CBF**		
	Acc	Indet	Unacc	Acc	Indet	Unacc
1	20	0	0	19	1	0
2	20	0	0	19	1	0
3	20	0	0	20	0	0
4	20	0	0	18	2	0
5	20	0	0	17	3	0
6	20	0	0	20	0	0
7	20	0	0	20	0	0
**Sum**	**140**	**0**	**0**	**133**	**7**	**0**

Table S.4: Comparison of responses for RAPID and MAGIC CTP on Question 2: Counts of Acceptable (Acc), Indeterminate (Indet), and Unacceptable (Unacc) ratings for MTT maps. Most images were rated "Acceptable" for both methods.

**Doctor**	**RAPID MTT**			**MAGIC MTT**		
	Acc	Indet	Unacc	Acc	Indet	Unacc
1	20	0	0	20	0	0
2	14	0	6	7	4	9
3	18	2	0	19	1	0
4	4	2	14	0	2	18
5	20	0	0	18	2	0
6	20	0	0	20	0	0
7	17	0	3	17	0	3
**Sum**	**113**	**4**	**23**	**101**	**9**	**30**

Table S.5: Comparison of responses for RAPID and MAGIC CTP on Question 2: Counts of Acceptable (Acc), Indeterminate (Indet), and Unacceptable (Unacc) ratings for TTP maps. Most images were rated "Acceptable" with few "Indeterminate" or "Unacceptable" responses.

**Doctor**	**RAPID TTP**			**MAGIC TTP**		
	Acc	Indet	Unacc	Acc	Indet	Unacc
1	19	1	0	19	1	0
2	18	2	0	15	4	1
3	20	0	0	18	2	0
4	14	5	1	14	6	0
5	20	0	0	18	2	0
6	20	0	0	20	0	0
7	18	0	2	20	0	0
**Sum**	**129**	**8**	**3**	**124**	**15**	**1**

Table S.6: Results for responses to Q3: "What diagnosis would be given to this patient based on their NCCT and CTP imaging?" The matrix on the left shows the distribution of diagnostic ratings (1–4) assigned to each case using real CTP (RAPID) versus synthetic CTP (MAGIC). The majority of responses are concentrated along the diagonal, indicating high agreement between diagnoses made with real and synthetic CTP images. For example, 43 cases were rated as category 1 by both methods, and similar agreement is seen for other categories.

**Method**		**MAGIC**			
	**Option**	**1**	**2**	**3**	**4**
**RAPID**	**1**	43	14	0	2
	**2**	13	9	2	6
	**3**	3	2	6	13
	**4**	6	2	1	16

Table S.7: Results for responses to Q4: "Rate your confidence in the above diagnosis." The matrix on the right displays the confidence ratings (1–5) for each case, comparing real and synthetic CTP. Most responses are clustered along the diagonal, especially at higher confidence levels (e.g., 44 cases rated as 4 by both methods, and 25 cases rated as 5 by both), indicating that raters' confidence in their diagnoses was generally consistent between RAPID and MAGIC CTP images.

**Method**		**MAGIC**				
	**Option**	**1**	**2**	**3**	**4**	**5**
	**1**	1	0	1	0	0
	**2**	0	3	1	4	2
**RAPID**	**3**	0	1	4	8	1
	**4**	0	9	6	44	15
	**5**	0	1	3	11	25

Table S.8: Ablation study results for the MAGIC model across four perfusion maps: Cerebral Blood Flow (CBF), Cerebral Blood Volume (CBV), Mean Transit Time (MTT), and Time to Peak (TTP). SSIM and UQI scores are reported for the full MAGIC model and for variants with specific loss components removed (no multimodal loss, no extrema loss, and no loss components). The results highlight the importance of each loss in contributing to the final performance.

Model	CBF		CBV		MTT		TTP	
	SSIM	UQI	SSIM	UQI	SSIM	UQI	SSIM	UQI
No-multimodal	0.7911	0.6129	0.7861	0.6222	0.8103	0.7422	0.79716	0.7203
No-both	0.7936	0.6186	0.7886	0.6243	**0.8130**	0.7442	0.80007	0.7234
No extrema	0.7835	0.6148	0.7807	0.5454	0.8036	0.7529	0.7997	0.7739
**MAGIC**	**0.8302**	**0.9313**	**0.8158**	**0.9037**	0.7938	**0.9453**	0.7975	**0.9066**

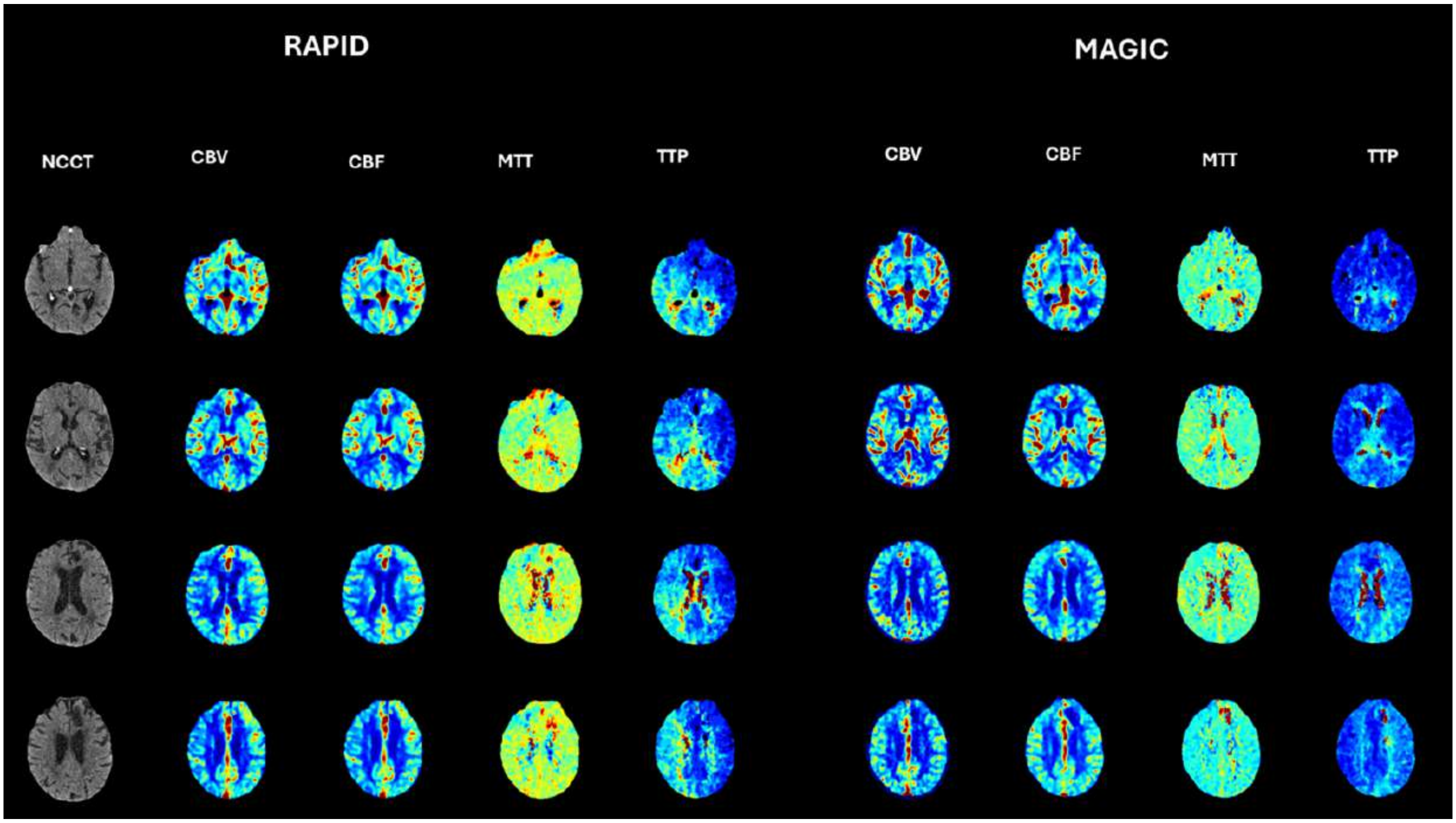

Figure S.4: Example of small-vessel ischemic injury on non-contrast CT imaging. This figure demonstrates the substantial low density of the periventricular white matter, especially near the occipital horns, which is typically indicative of small vessel ischemic injury. Despite this, the perfusion parameters measured in the current MAGIC iteration do not reflect this condition, highlighting a limitation of the proposed method in detecting small-vessel occlusions based solely on non-contrast imaging.

Table S.9: Comparison of MAGIC with state-of-the-art models (U-Net and Pix2Pix) across CBF, CBV, MTT, and TTP.

**Model**	**CBF**		**CBV**		**MTT**		**TTP**	
	SSIM	UQI	SSIM	UQI	SSIM	UQI	SSIM	UQI
UNet	0.6164	0.7747	0.7951	**0.9093**	0.8308	0.8783	0.8105	0.8600
Pix2pix	0.7561	0.4519	0.8068	0.8828	**0.8320**	0.8353	**0.8176**	0.8152
MAGIC	**0.8302**	**0.9313**	**0.8158**	0.9037	0.7938	**0.9453**	0.7975	**0.9066**

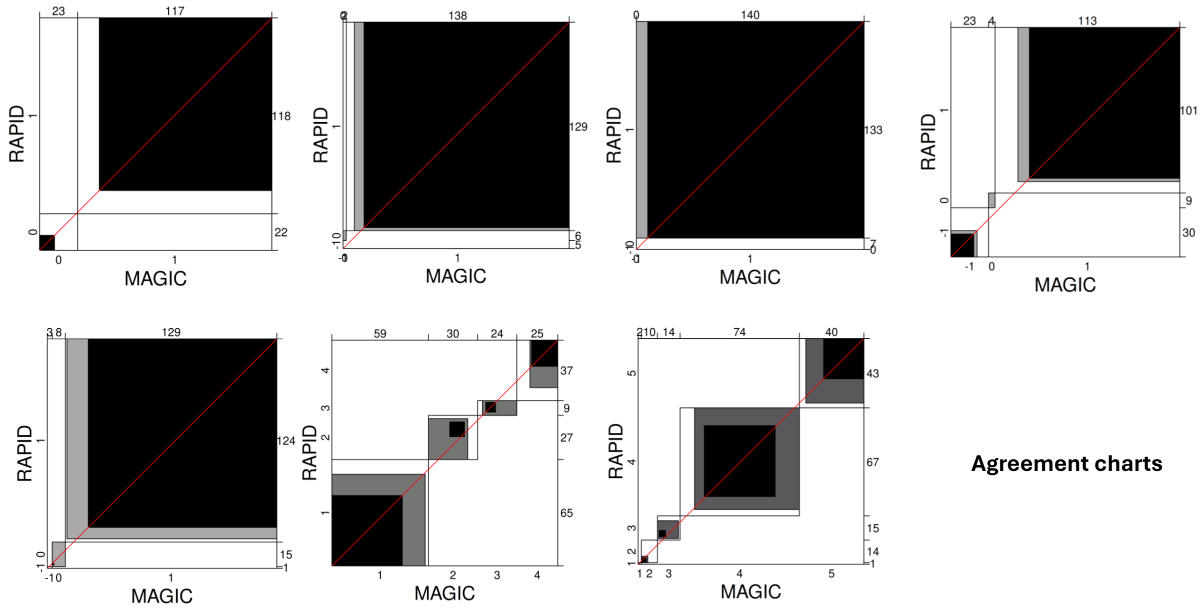

Figure S.5: Agreement plots based on contingency tables. The first row shows four plots from left to right: Q1, Q2A, Q2B, and Q2C. The second row shows three plots from left to right: Q2D, Q3, and Q4.
